# Five years later, with double the demographic data, naked mole-rat mortality rates continue to defy Gompertzian laws by not increasing with age

**DOI:** 10.1007/s11357-024-01201-4

**Published:** 2024-05-21

**Authors:** J. Graham Ruby, Megan Smith, Rochelle Buffenstein

**Affiliations:** 1 Calico Life Sciences LLC, 1170 Veterans Blvd, South San Francisco, CA 94080 USA; 2 Department of Biological Sciences, University of Illinois, Chicago 845 W Taylor, Chicago, IL 60607 USA

**Keywords:** Demographic aging, social dynamics

## Abstract

The naked mole-rat (*Heterocephalus glaber*) is a mouse-sized rodent species, notable for its eusociality and long lifespan. Previously, we reported that demographic aging, i.e., the exponential increase of mortality hazard that accompanies advancing age in mammals and other organisms, does not occur in naked mole-rats (Ruby et al., 2018), a finding that has potential implications for human healthy aging. The demographic data supporting that conclusion had taken over three decades to accumulate, starting with the original rearing of *H. glaber* in captivity. This finding was controversial since many of the animals in that study were relatively young. In the 5 years following that study, we have doubled our quantity of demographic data. Here, we re-evaluated our prior conclusions in light of these new data and found them to be not only supported but indeed strengthened. We additionally provided insight into the social dynamics of captive *H. glaber* with data and analyses of body weight and colony size versus mortality. Finally, we provide a phylogenetically proximal comparator in the form of lifespan data from our Damaraland mole-rat (*Fukomys damarensis*) colony and demographic meta-analysis of those data along with published data from Ansell’s mole-rat (*Fukomys anselli*). We found *Fukomys* mortality hazard to increase gradually with age, an observation with inferences on the evolution of exceptional lifespan among mole-rats and the ecological factors that may have accompanied that evolution.

**Supplementary Information:**

The online version contains supplementary material available at 10.1007/s11357-024-01201-4.

## Introduction

The species *Heterocephalus glaber*, commonly known as the naked mole-rat, is a eusocial mammal endemic to the arid and semi-arid regions of northeast Africa. In the wild, naked mole-rats live an almost completely subterranean lifestyle, in colonies of up to 295 animals (average size is 60 animals/colony [1]) that cohabitate a network of tunnels that the mole-rats dig themselves with their large, procumbent ever-growing incisors [2]. Each colony is usually led by a single, socially dominant queen (the breeding female), living with a small number of breeding males and a large number of non-breeding progeny. New breeding females arise either through conflict following the death of a queen or through isolation of part of the colony, e.g., through tunnel collapse [2–4].

Naked mole-rats are notable for their extreme lifespans [5], living longer than any other documented rodent, with their longest previously reported lifespan of 37 years [6]. More recently, the oldest animal in our care lived to be 40 years old and many other animals in our colonies are living beyond 30 years [7, 8]. These values are notable in the context of this species’ small body size due to the strong correlation across species between body size and mammalian lifespan: maximum lifespan potential (MLSP) increases by 16% for each doubling of average species body mass [9]. Notably, naked mole-rats live approximately five times longer than their size-corrected, allometric expectation [10]. For most mammalian species, lifespan is limited by an exponential increase in the per-day risk of death (i.e., mortality hazard) with age [11, 12] in accord with a statistical distribution first defined by Gompertz based on human mortality [13]. The increase in mortality hazard with age is referred to as “demographic aging.” We have previously shown that *H. glaber* achieves its exceptional longevity through defiance of this trend, exhibiting ~constant mortality hazard across the full spectrum of observed lifespans, with no evidence of hazard increase even at ages many times greater than their expected MLSP [14, 15].

Naked mole-rats are members of the African mole-rat superfamily, Bathyergidae, that includes more than 30 species of subterranean rodents. Naked mole-rats appeared to split from the other bathyergids ~26 million years ago (MYA) [16, 17]. Bathyergid species span the spectrum of social behavior, from solitary (e.g., *Georychus*, *Heliophobus*, and *Bathyergus*) to the only other known eusocial mammals, members of the genus *Fukomys* [18]. Along with eusociality, *H. glaber* also have in common with members of the *Fukomys* genus (namely *Fukomys damarensis* and *Fukomys anselli*) above expected longevity, living ~twice as long as their size-corrected, allometric expectations (see the “Methods” section for details). The co-occurrence of these unusual properties in divergent mole-rat clades motivates a question about the similarity of age-related hazard patterns.

For naked mole-rats, the lack of demographic aging is accompanied by seemingly indefinite maintenance of many physiological characteristics that typically start changing before attaining species median lifespan. Naked mole-rats are relatively resistant to age-related diseases such as cancer, neurodegeneration, and cardiovascular disease [8, 19, 20] and show signs of tissue regeneration and remodeling preventing the deterioration of age-associated physiological function [2, 8, 21, 22]. Despite well-maintained cardiac [8] and metabolic function [23], we have previously reported that there are some signs of age-related changes in activity and morphology (such as paler skin tones) in some, but not all, older animals [19, 24, 25]. Indeed, we reported that in two necropsy-based studies of 29-year-old animals, there were signs of sarcopenia, osteoarthritis, and cataracts [19]. However, this and other studies also report that the great majority of animals remain active, continue to breed well into their third decade, show no menopause, and have unchanged muscle and fat mass as well as metabolic profiles [21–24, 26–28]. The health deteriorations that we have reported in naked mole-rats (e.g., cataracts and osteoarthritis) appear to change neither in severity nor in frequency as age increases: for example, cataracts have been observed in juveniles (<3 months old) and adults in every decade of life; similarly, common age-related pathologies are evident across all age cohorts [29]. Rather, death and disease both appear to be stochastic and independent of chronological age, consistently supporting the classification of the naked mole-rat, at least until their third decade of life, as a “non-aging mammal” [14]. There are also several indications of maintenance of neotenous traits throughout the life of *H. glaber* [21, 30]. Nevertheless, there is also some molecular evidence for chronological aging as seen in the epigenetic (DNA methylation) clock, albeit showing that the longer-lived naked mole-rat breeders maintain a more youthful methyl clock [31]. There are several other genomic features associated with attenuated aging, such as positive selection of *TERF1* protecting telomeres [32] as well as high expression levels of genes involved in DNA repair [33].

Our earlier paper [14] reporting no observation of the onset of actuarial senescence in the naked mole-rat stoked considerable controversy. A commentary on our original demographic analysis asserted that loss of records from our early years of our vivarium rearing of *H. glaber* could have confounded our analysis of aging demographics [34]. The concern of that critique was addressed using left-censorship [15]. However, the critique also highlighted the unique nature of our collection as a resource for powered demographic analysis of naked mole-rats, extending out to advanced ages. The lack of other collections of a similar scale, worldwide, presents a practical challenge to the reproducibility of our results. To that end, we were motivated to re-evaluate our original conclusions, using demographic data for *H. glaber* collected over a 5-year period since the data freeze of our earlier paper [14].

Here, we re-visited the demographic analysis of our naked mole-rat collection, with husbandry data now extended by 5 years across an expanded set of animals. Over the four-decade range of animal ages in our colony, we found our original conclusions [14] of naked mole-rat mortality hazard being age-independent to be reproduced, regardless of whether or not we used either the total sum of all historical data or only those data collected after our previous study—the latter qualifying as a replication study. We further conducted an analysis of naked mole-rat mortality hazard versus colony size which suggested that social competition was greater for non-breeders in smaller colonies. Finally, we provide demographic data for Damaraland mole-rats: also combining those data with other publicly available data for *Fukomys* species [34], we analyzed age-specific mortality hazard and showed it to increase, in contrast to the naked mole-rats.

## Materials and methods

### Animal husbandry

Husbandry practices for *H. glaber* were identical to those described previously [14]. A similar husbandry protocol was used for the Damaraland mole-rats.

The original progenitors of our Damaraland mole-rat colony were trapped near the town of Hotazel, South Africa (27°58′S, 17°41′E), and near the town of Dordabis in Namibia (22°58′S, 17°41′E). Like the naked mole-rat, all animals were microchipped at 90 days with a unique RFID number. Similarly, Damaraland mole-rats were group-housed in interconnected transparent multi-cage systems, joined together by plexiglass clear tubing in a climatically controlled room maintained at 26–28°C, 21% oxygen, and with RH ranging between 20–50% and with a 12L:12D light cycle. Animals were fed daily a variety of fresh fruit and vegetables, including apples, yams, carrots, pumpkin, and other seasonal vegetables, replacing the natural geophytes upon which they feed in the wild. No water was provided as the animals meet all their water requirements through their diet or metabolic water production.

### *H. glaber* demographic data

Demographic analysis of *H. glaber* was performed on a newly compiled aggregation of lifespan data, from across the collection of naked mole-rats described in [14], which had been maintained and expanded under the same husbandry conditions described there. For this study, a snapshot of records was compiled on March 15, 2021, at which point they contained information for 7536 animals from across the full history of the collection. Of those, complete birth and death/right-censorship information (single-day resolution) were available for 6949 animals. For those animals, and versus our prior snapshot of records from this collection—–provided as “Supplementary data and Supplemental Table 1” in [14]—3329 could be matched to the prior records. Of those, 2806 had perfect matches to RFID tags, birth dates, sex, breeder status, colony name, and logically consistent death/censorship data (for animals alive at the prior snapshot, new death/censorship dates from after those dates; otherwise, perfect matches). For an additional 94 animals, all data listed above were identical except for either colony name or breeding status: both of these parameters are expected to change for animals moved out of a colony to establish a new colony as a founding breeder. Combined, 3222 animals matched for all vital data (RFID, birth date, sex, and consistent death/censorship). For an additional 94 animals, one of the four vital data had been updated: 11 for RFID (consistent with occasional replacement of RFID chips, or placement of RFID chips in previously un-chipped animals); 32 for sex (consistent with inaccurate initial sex determination for young animals); 40 for date of birth (consistent with review of birth data at the time of RFID implantation); and 11 with updated and inconsistent death/censorship dates. For all 94 of those animals, the updated values were used for analysis. For an additional five animals, two pieces of vital data differed: one had inconsistent death/censorship and sex; one had inconsistent death/censorship and date of birth; and three had different dates of birth and sexes. For those animals as well, the updated values were used for analysis. For an additional eight animals, exact dates of death/censorship were missing from current records but found in the prior snapshots, with all other vital data matching. For those animals, the death/censorship values and dates from the [14] snapshot were used. An additional 26 animals had RFID matches to animals that had missing or low-resolution birth and/or death/censorship data in our earlier snapshot [14]; those animals were assigned their original table ID’s and current data values. In total, 3355 animals were paired to individuals cataloged in “Supplemental Table 1” of [14]; all of those are presented in Supplementary Data using their original ID’s (e.g., “Hg-02241”).

An additional 3602 animals with day-resolution birth and death/censorship data were newly added to the roster presented in Supplementary Data and Supplemental Table 1, versus the catalog from [14]. Those animals were all assigned ID numbers ≥5000 (e.g., “Hg-05072”). The vast majority of those animals were born after (3244) or during (33) the prior data consolidation period (April 14–May 17, 2016). Of the remaining 325 animals, the large majority (285) were born within a year prior to April 14, 2016, reflecting a normal lag for husbandry records to be entered into our database.

In summary, 6949 naked mole-rats were cataloged with day-resolution birth and death/censorship data, 3355 of which could be matched to animals from [14]. An additional eight animals with missing death/censorship data in the current inventory were included using those published data from [14], bringing the total number of animals analyzed to 6957. For still-surviving animals, right censorship was applied at the date of record aggregation: March 15, 2021.

### Analysis of lifespan and age-specific mortality hazard

Except as otherwise specified in the text, right censorship status was applied to animal lifespans in three cases: if the animal was (a) transferred to another collection, with the date of transfer considered as the censorship event; (b) euthanized for research purposes when still healthy, with the date of sacrifice as the censorship event; or (c) still alive when the process of compiling records was completed, with the date of completion as the censorship event. Left censorship was applied to the entire data sets as specified in the text, generally at specific historical dates in order to exclude data collected prior to that date.

Kaplan-Meier survival was calculated using their method [35]. Mortality hazards were calculated for each specified age bin/group of animals as the number of observed death events (i.e., number of days on which a naked mole-rat was observed to have died) divided by the total number of days that naked mole-rats were under observation, limited to that age bin/population. Confidence intervals were calculated using the Wilson score interval with continuity correction [36].

Regarding left censorship and truncation, as reviewed in [37], Kaplan-Meier survival analysis provides a robust method that is inclusive of data from individuals who either enter a study after the initiating event (left-censored individuals) or do not experience an endpoint event until after either the end of the study or their exit from the study for irrelevant reasons (right-censored individuals). That robustness also applies to life-table/actuarial analysis [38], which we also employ. Survival analyses can be biased by data truncation, i.e., non-inclusion of time-to-event data when an endpoint is reached prior to study initiation [39]. The potential for such bias in our original analysis [14] was pointed out in a commentary [34], and we addressed this using left-censorship, i.e., limiting the use of survival data to a time period where we could be confident that lifespan data from all individuals was available [15]. Kaplan-Meier survival analysis also assumes that censored and uncensored individuals have the same survival probabilities, and that the survival probabilities are similar for individuals recruited (here, reaching *T*_sex_) early or late in the study [37]. While unlikely to affect the conclusions of our prior analysis [14], such bias is possible due to the long time period across which our data were collected. In the current study, we allayed concerns of such bias through the observation of similar results from independent analysis of survival data collected during two non-overlapping periods (before and after the data freeze data from our prior study; see the “Results” section).

### *H. glaber* body weight analysis

Historical records of body weights, collected in the course of animal health checks, were aggregated concurrently with the lifespan data from [14]. These records were less complete than the demographic data, but nonetheless included 19,118 measurements taken from 1940 animals with complete lifespan data. For most analyses, only weights taken after 500 years of age were used: there were 6785 such measurements, taken from 942 animals. The demographic data used for these analyses was as compiled previously [14], and it is provided along with colony membership and body weight data in Supplemental Table 2. Animals from [14] with full demographic data but no body weight data are also included in that file. Animal IDs in that file are matched to our earlier study [14] and Supplemental Data File 1.

For mortality versus body weight, analysis was performed for survival across a single year. For each animal with body weight(s) measured after 500 days of age, survival data were analyzed starting from the date of the last recorded body weight (i.e., left-censored on that date). If the animal survived uncensored for over a year, then right-censorship was applied 1 year after the weight measurement. Animals with qualifying body weight values were sorted into quartiles based on those final measurements, and mortality hazard was estimated across all of the animals in each quartile. Confidence intervals were calculated using the Wilson score interval with continuity correction [36].

### *H. glaber* colony size analysis

For mortality versus colony size, analyses were performed of survival across a single year. The results for each year are shown in Supplementary data and Supplemental Table 1 and Supplemental Figure S1: for years 2012–2015, demographic records from [14] (also provided in Supplemental Data File) were used, and April 14 was used as the cutoff date; for years 2016–2020, the updated demographic records from this manuscript were used (Supplemental Tables 1-3), and March 15 was used as the cutoff date. Cutoff dates were selected to match the data compilation date for each data set, ensuring a full year’s worth of data availability for each year’s analysis. The shift to an older record compilation for earlier-year analyses was to minimize the effect of movement out of colonies as a confounder: colony affiliations for each compiled data set reflected colony membership at the time of compilation and did not include prior membership history. Importantly, this caveat should have had minimal impact: due to violence against intruders, animals are never introduced into existing colonies, only removed from colonies in order to establish new colonies as breeders [40], and breeders were excluded from these analyses.

For analysis of colony sizes, only animals at least 500 days old by each cutoff date were considered. This decision was justified by animals younger than that age being not fully grown and therefore potentially presenting less competitive rivalry, and by the inconsistent integration of animals much younger than 1 year (pre-chipped) old into our demography database. But it may have added variance in the form of colony-size error in the cases of colonies with highly skewed age distributions. Analyses were also conducted in a sex-specific manner, i.e., considering only the counts of same-sex, non-breeding animals for colony size. This decision was justified under the assumption that competition and hierarchy may be different between the two sexes [41], but may have added variance in cases where the sex ratio was skewed in a colony, to the extent that social hierarchy among non-breeders is sex-independent.

For each cutoff date, for each sex, all qualifying animals were used to generate a census (animal count) for each represented colony name. Individual animals were sorted into quartiles based on the sizes of the colonies of which they were members; tied animals were assigned to adjacent quartiles arbitrarily. For members of each quartile, the mean and standard deviation of colony sizes for each animal were calculated, and the mortality hazard for those animals was calculated across 1 year, beginning at the cutoff date (i.e., left-censored on the cutoff date, and right censored 1 year after the cutoff date for animals that were not deceased or otherwise censored during the year). Confidence intervals were calculated using the Wilson score interval with continuity correction [36].

For regression of mean colony size versus mortality hazard, for each sex: for all quartiles across all years shown in Supplemental Figure S1, mean colony sizes were treated as *x*-axis values and mortality hazard estimates as *y*-axis values, and linear regression was performed using the “scipy.stats.linregress” function from Scipy [42], which in addition to slope and intercept returns Pearson R and regression *p*-value.

### *F. damarensis* demographic data

As for *H. glaber*, lifespan data from our longstanding collection of Damaraland mole-rats, *F. damarensis*, were compiled on March 15, 2021, with that date used as the right-censorship point for still-living animals. Of 754 animals with records, 702 had day-resolution birth and death/censorship data and were therefore included in these analyses. Per-animal demographic data are provided in Supplemental Data File 3.

### Calculation of maximum versus observed expected lifespan

Allometric expectations were determined using the equation from de Magalhães [10]:

$${T}_{\textrm{max}}=4.88\ast {m}^{0.153}$$

where body mass *m* is in grams and *T*_max_, predicted maximum lifespan, is in years. The longevity quotient (LQ) was defined as the ratio of observed MLSP to *T*_max_. For *H. glaber*, using a body mass = 35g, *T*_max_ = 8.4 years, and with observed MLSP = 40 years, LQ = 4.8. For *F. anselli*, using a body mass = 90g, *T*_max_ = 9.7 years, and with observed MLSP = 22 years, LQ = 2.3. For *F. damarensis*, using a body mass = 160g, *T*_max_ = 10.6 years, and with observed MLSP = 20 years, LQ = 1.9.

## Results

### The expanded data set revealed that the hazard of naked mole-rat mortality did not increase as a function of age

**Fig. 1:**
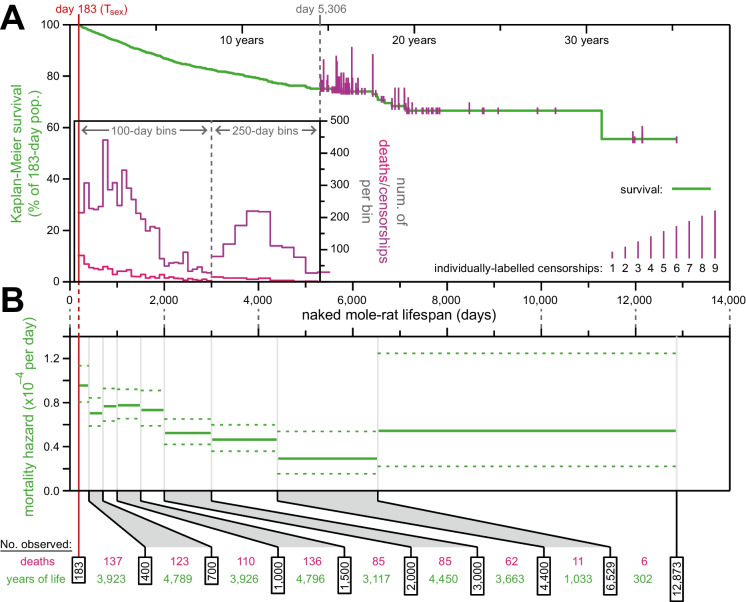
The mortality hazard of naked mole-rats failed to increase with age. **A** Kaplan-Meier survival curve for naked mole-rats (green) after reaching reproductive maturity (*T*_sex_; 6 months from birth; 183 days; red). Plotted as in Fig. 1A of Ruby et al. (2018), with a histogram of death and censorship (pink and purple, respectively) in the inset, up until 5306 days of age, at which point 174 animals remained in the population; and with censorship events after that indicated by vertical ticks (purple), the size of which is proportional to the number of animals censored at each day-of-life. **B** Mortality-hazard estimates (solid green) with 95% confidence intervals (dotted green) across the observed naked mole-rat lifespan. Estimates were calculated across time intervals of increasing size (demarked by grey lines) to compensate for decreasing accuracy per unit time as the population size decreased. The borders of the age bins (black), along with the number of deaths (pink) and total years of animal life observed (green) per bin, are indicated at the bottom.

Our expanded data set drew from 7536 historically catalogued naked mole-rats from our own collection (Supplementary data file and Supplemental Tables 1–3); of these, 6957 had sufficiently high (single-day) resolution records for date-of-birth and date-of-death/censorship to be included (see the “Methods” section for details). Our analyses of survival and mortality hazard began at *T*_sex_ for naked mole-rats: 6 months (183 days [19]); 6893 animals had aged up to *T*_sex_ and therefore were included in our downstream analyses. Of those, 755 had recorded deaths and 6138 were right-censored (see the “Methods” section for censorship criteria). Kaplan-Meier survival, along with death and censorship distributions, are plotted in Fig. 1A. The last recorded death was at 11,281 days-of-life (30.9 years), at which point 10 animals remained alive; the last of those was censored at 12,873 days-of-life (35.2 years). At that event, Kaplan-Meier survival remained at 55.5%. One cannot emphasize enough that at ages almost an order of magnitude greater than that of similar sized mice, naked mole-rats have not yet reached their median lifespan.

Using the survival data, we calculated age-specific mortality hazard across the same age-range windows as in [14] (Fig. 1B). As in the prior analysis, mortality hazard estimates remained consistently low across the ~35 years of age encompassed by the observations, never exceeding a per-day probability of 1/10,000. This very low risk of dying in our colonies likely reflects that we have optimized our animal husbandry protocols reducing not only the risk of dying due to extrinsic factors but also due to potential dietary deficiencies and unstable colony dynamics.

### Analysis of only data collected after our prior survey independently confirmed constant-hazard demography

**Fig. 2:**
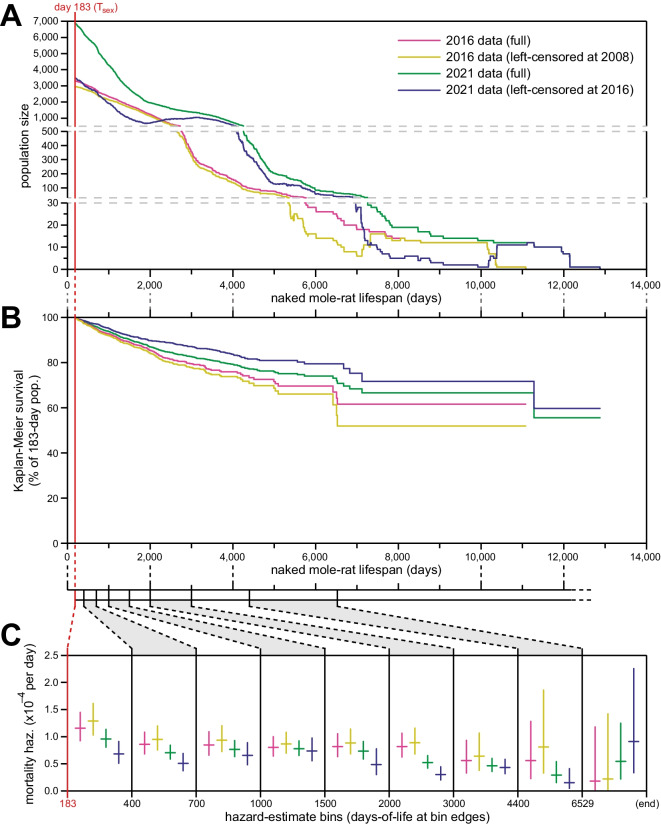
Exclusion of neither pre-2008 nor pre-2016 lifespan data through left-censorship modified the observed lifespan demographics of *H. glaber*. **A** The number of naked mole-rats observed (*y*-axis) at each age (*x*-axis) for four datasets: (i) the original data set presented in (Ruby et al., 2018) (pink); (ii) that data set left-censored on January 1, 2008, as presented in (Ruby et al., 2019) (yellow); (iii) the full data set compiled in 2021 for this manuscript (green); (iv) that data set left-censored on May 17, 2016 (navy), i.e., data collected after the compilation of data for (Ruby et al., 2018). **B** Kaplan–Meier survival curves starting at *T*_sex_ (183 days; red) for the four demographic data sets, as colored and described in panel **A**. **C** Mortality hazard estimates (*y*-axis) across each of the lifespan bins from panel **B** (indicated on the *x*-axis), for each of the four demographic data sets, as colored and described in panel **A**. Horizontal bars indicate hazard estimates, and vertical bars indicate 95% confidence intervals.

The large size of our naked mole-rat collection across the 5 years of additional observation time since our previous report [14] provided substantial additional power in the form of number of animals observed at most ages (Fig. 2A). For many age groups, the amount of lifespan observation time since our previous snapshot matched or exceeded that prior to it. This allowed us to use left-censorship at a date after our original dates of record aggregation to perform an independent analysis of naked mole-rat lifespan demographics and critically re-evaluate our original conclusions.

For our previous report, lifespan records were aggregated in three batches: on April 14, 2016; April 20, 2016; and May 17, 2016 [14]. Animal counts as a function of age, Kaplan-Meier survival, and age bin-specific hazard are shown for that data set in magenta in Fig. 2A, B, and C, respectively. The possibility that incomplete record survival during the earliest years of our keeping naked mole-rats in captivity could have skewed mortality hazard trends was raised by [34], and we previously addressed that concern by excluding data from those early years of naked mole-rat captivity, via left-censorship on January 1, 2008 [15]. No additional data were provided at that time. The scale, Kaplan-Meier survival, and age-specific hazard from that left-censored version of our original data set are shown in orange in Fig. 2A, B, and C, respectively.

The scale, Kaplan-Meier survival, and age-specific hazard from our current, full data set—also depicted in Fig. 1—are shown in green in Fig. 2A, B, and C, respectively. These data were aggregated on March 15, 2021, and encapsulated all historical data. In order to perform an analysis of naked mole-rat lifespan demographics that would be independent of our original analysis, we left-censored these data on May 17, 2016. The scale, Kaplan-Meier survival, and age-specific hazard from this left-censored data set are shown in blue in Fig. 2A, B, and C, respectively. For each age bin, the new, left-censored hazards were either lower or statistically indistinguishable from the original data set, either full or left-censored (Fig. 2C). We therefore maintain our original conclusion: that unlike every other species studied to date where mortality risk begins to increase long before median lifespan, naked mole-rat mortality hazard does not increase with age, with that assertion being statistically robust to the beginning of our final estimate bin (6529 days, or ~18 years), an age many-fold greater than their expected maximum lifespan.

### Non-increasing mortality hazard was exhibited by breeders and non-breeders of both sexes

**Fig. 3:**
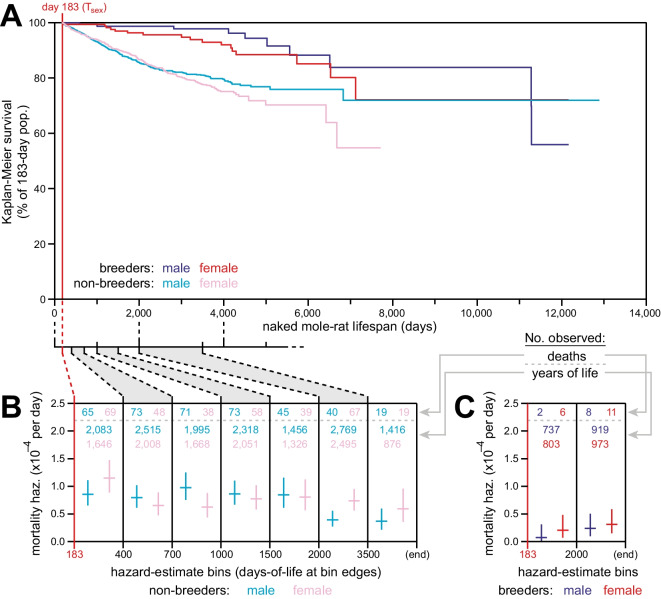
Breeding and non-breeding male and female naked mole-rats exhibited non-increasing mortality hazard as a function of age. **A** Kaplan-Meier survival starting at *T*_sex_ (183 days; red) for each of four reproductive categories of naked mole-rats: male breeders (navy), female breeders, (red), male non-breeders (cyan), and female non-breeders (pink). **B** Mortality hazard estimates (*y*-axis) for the indicated lifespan bins (*x*-axis), calculated for non-breeding males (cyan) and females (pink). The numbers of observed death events per bin are indicated at the top of each bin. **C** Mortality hazard estimates (*y*-axis) for the indicated lifespan bins (*x*-axis), calculated for breeding males (navy) and females (red). The numbers of observed death events per bin are indicated at the top of each bin.

The analyses described above were performed on our full population of animals. Sub-populations defined by sex and breeding status were analyzed individually for Kaplan-Meier survival (Fig. 3A). As previously observed [14], there was a notable survival difference between breeding versus non-breeding status, with breeders enjoying longer survival than non-breeders. Any individual is capable of becoming a breeder and in captivity, many of the animals given the opportunity to breed are randomly chosen, isolated from their colony and subsequently paired. As such, it is unlikely that only “high-quality” individuals become breeders and that this is what drives the disparate mortality risks between breeders and non-breeders.

As with the aggregate population, mortality hazard remained consistent with age, for both non-breeders (Fig. 3B) and breeders (Fig. 3C), with the mortality hazard of breeders only ~20% that of non-breeders. As previously observed, male non-breeders exhibited enhanced survival (Fig. 3A) and reduced mortality hazard (Fig. 3B) at ages beyond 2000 days. This concurred with the expectations of our model that multiple males can enjoy breeder status in a colony despite not being labeled as such (only the founding breeders are so labeled [14]).

### Non-breeder mortality hazard was influenced by colony size

Some of the mortality hazard difference between breeders and non-breeders may derive from social struggle between these hierarchies within the colony. To explore the potential effects of intra-colony social dynamics on mortality hazard, we performed analyses of the relationships between body weight or colony size on the mortality of non-breeders.

**Fig. 4:**
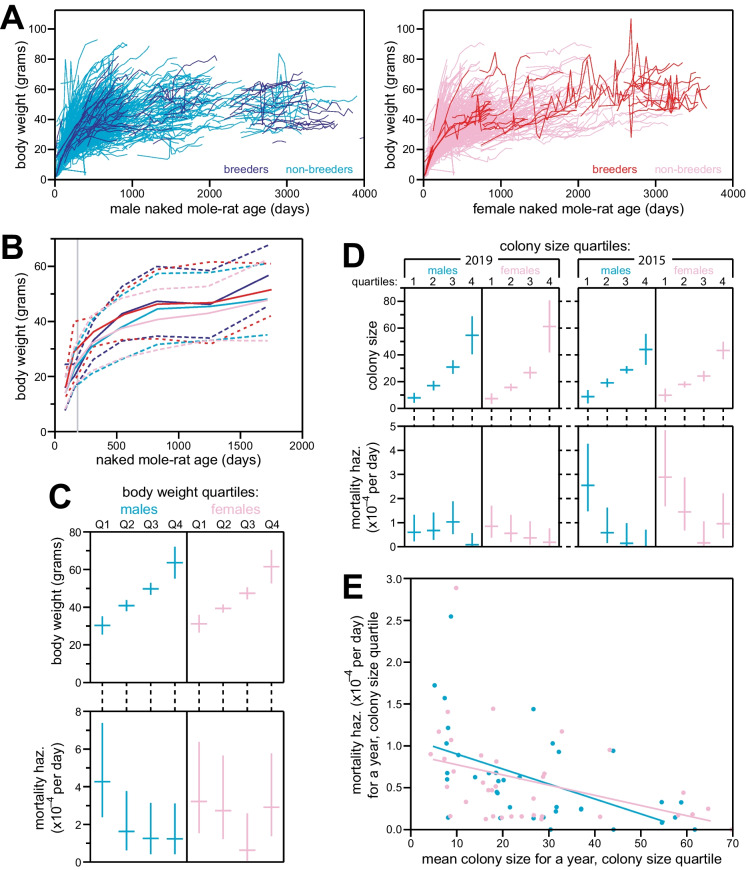
Non-breeding naked mole-rat mortality hazard decreased with increasing body weight and colony size. **A** Individual body weight values (*y*-axis) plotted longitudinally versus age (*x*-axis) for breeding males (navy), non-breeding males (cyan), breeding females (red), and non-breeding females (pink). **B** Mean body-weights for each of the categories from panel **A**, for all weight measurements taken across each of the following age bins: 50–120 days, 120–200 days, 200–400 days, 400–700 days, 700–1000 days, 1000–1500 days, and 1500–2000 days; inclusive of the 1^st^ day and non-inclusive of the last day for each bin. Solid lines indicate mean body weights; dotted lines indicate standard deviations. **C** Top panel: body weight quartiles for male (left) and female (right) non-breeding animals, organized based on the last recorded weight measurement for each animal and only included if that measurement was taken at an older age than 500 days. Horizontal bars indicate the mean body weight for each quartile; vertical bars indicate standard deviations. Bottom panel: mortality hazard for each body weight quartile in the top panel, calculated for the year following weight measurement. See the “Methods” section for details. Horizontal bars indicate mortality hazard estimates; vertical bars indicate 95% confidence intervals. **D** Examples of colony-size versus mortality-hazard plots. For each indicated year (2019 on the left; 2015 on the right), and for each indicated non-breeding sex (males on the left; females on the right): animals were organized into quartiles based on the number same-sex, non-breeding animals in their colony. Top panels: the mean colony sizes for animals in each quartile; vertical bars indicate standard deviations. Bottom panels: mortality hazard estimates for each colony-size quartile from the top panel; vertical bars indicate 95% confidence intervals. Similar analyses were performed for each year, 2012–2020; all plots are provided in Supplemental Figure S1. See the “Methods” section for details. **E** For all colony-size quartiles, from all years analyzed in Supplemental Figure S1: a meta-analysis of quartile mean colony sizes (*x*-axis) versus 1-year mortality hazards (*y*-axis). Non-breeding males (cyan) and females (pink) were meta-analyzed separately. Regression lines for each sex are shown (*p*-values, 9.8 × 10^−4^ for males, 0.018 for females).

For body weights, we analyzed a data set that had been aggregated to accompany our originally published demographic data [14] (Supplementary Data file and Supplemental Table 2). These data included 19,118 individual body weight estimates from 1940 animals, captured longitudinally for animals of a variety of ages, with data becoming sparse after ~3500 days of age (Fig. 4A). These data are provided in Supplemental Data File 2. A great deal of variation existed across both the breeder and non-breeder populations, in both males and females, though the age-specific averages were similar and stable after ~500 days of age (Fig. 4B).

For the analysis of mortality hazard versus body weight, only non-breeding animals were included. The last available body weight measurement was used, requiring that measurement was taken after 500 days of age, to avoid confounding with the social status of animals that were not yet fully grown. There were 6785 qualifying measurements, derived from 942 animals. For each animal, mortality hazard was analyzed across 1 year following that final measurement. Non-breeding animals of each sex were grouped into quartiles based on body weight, and the aggregate mortality hazard of each quartile is plotted, along with the aggregate body weight values, in Fig. 4C. There was a general trend of lighter/smaller animals having greater mortality hazard, especially in males, though the data set was insufficiently powered to statistically support this conclusion.

For colony sizes, we used our records’ annotations of “colony ID” to determine the number of non-breeding animals of each sex in each colony, at each analytical time point (Supplemental Table 1 and see Methods). As for body weight, animals were separated into quartiles based on the number of non-breeders of the same sex in the colony, and each quartile for each sex was cumulatively evaluated for mortality hazard across 1 year following the analytical time point. This method allowed independent analysis of non-overlapping time periods: we performed five such analyses using our new data set and four using our prior data set, from [14] (examples in Fig. 4D; all results shown in Supplemental Figure S2). Analyses of individual time blocks were statistically inconclusive, but there was a general trend of greater mortality hazard for non-breeders in smaller colonies. Meta-analysis of the results from across all nine time-block analyses supported this trend to a statistically significant degree, for both males (*p*-value: 9.9 × 10^−4^) and, to a lesser degree, females (*p*-value: 1.8 × 10^−2^).

### The mortality hazard of mole-rats of the Fukomys genus increased substantially with age

We have previously placed the mortality demographics of naked mole-rats into the context of demographic data from other mammals, which highlights the unique absence of an exponential increase in hazard with age in naked mole-rats [14]. To provide a more phylogenetically proximal comparator, we performed a demographic analysis of mole-rats from the *Fukomys* genus. For those species, survival analyses all began at an estimated *T*_sex_ of 270 days of age, based upon our unpublished data and confirmed by personal communication from Nigel C. Bennett (MRI, University of Pretoria) and Christopher G. Faulkes (Queen Mary University of London).

In their response to our previous publication, Dammann [34] provide lifespan data for 339 *Fukomys anselli* individuals, of which 332 aged past *T*_sex_ and therefore were included in our downstream analyses (Supplemental Figure S2A: yellow). In their analyses of lifespan, they censor accidental deaths and deaths from fighting: in that case, 170 animals’ lifespans are censored and 162 died [34]. Here and previously [14], we treat such instances as non-censored deaths: in that case, 145 animals’ lifespans are censored and 187 had died. Comparing Kaplan-Meier survival analyses, the death versus censorship of these 25 individuals had a small effect (Supplemental Figure S2B: yellow versus orange), only reducing the median lifespan from 3141 to 2830 days, nor were any statistical differences observed for age-specific mortality hazard estimates effect (Supplemental Figure S2C: yellow versus orange). For downstream analyses, we considered those 25 individuals to be dead rather than censored.

**Fig. 5:**
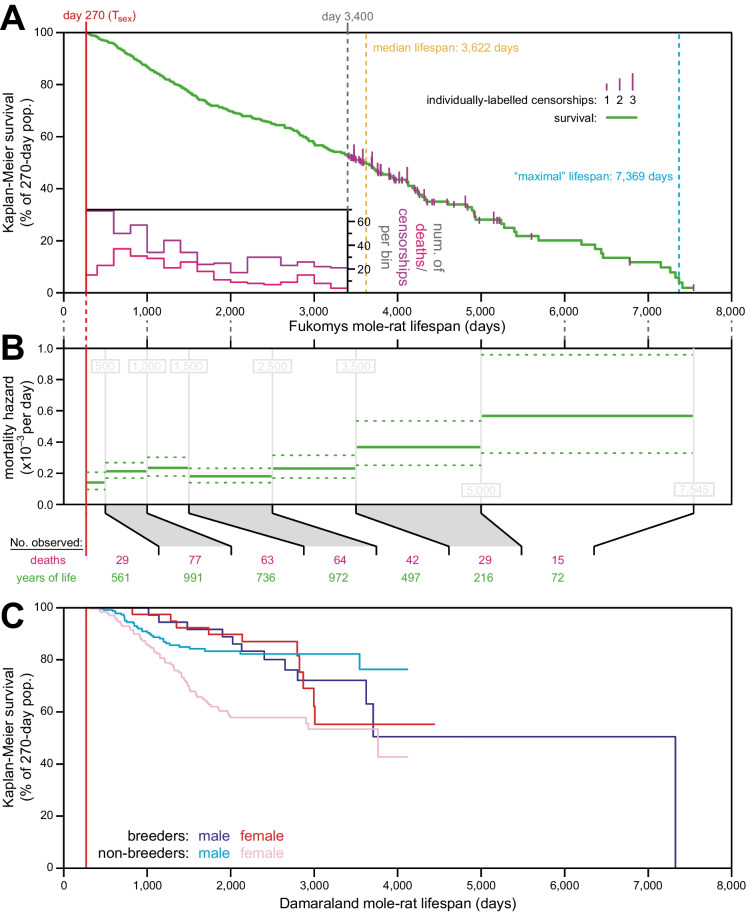
The mortality hazard of *Fukomys* mole-rats increased with age. **A** Kaplan-Meier survival curve for combined data from the *F. anselli* and *F. damarensis* species of mole-rats (green) after reaching reproductive maturity (*T*_sex_; 9 months from birth; 270 days; red). Plotted as in Fig. 1A, with a histogram of death and censorship (pink and purple, respectively) in the inset, up until 3400 days of age, at which point 120 animals remained in the population; and with censorship events after that indicated by vertical ticks (purple), the size of which is proportional to the number of animals censored at each day-of-life. Equivalent per-species analyses are provided in Supplemental Figure S2. **B** Mortality-hazard estimates (solid green) with 95% confidence intervals (dotted green) across the observed *Fukomys* mole-rat lifespan. Estimates were calculated across time intervals of increasing size (demarked by grey lines) to compensate for decreasing accuracy per unit time as the population size decreased. The borders of the age bins are indicated in gray, and the number of deaths (pink) and total years of animal life observed (green) per bin are indicated at the bottom of the panel. Equivalent per-species analyses are provided in Supplemental Figure S2. **C** For *F.damarensis* only: Kaplan-Meier survival starting at *T*_sex_ (270 days; red) for each of four reproductive categories of Damaraland mole-rats: male breeders (navy), female breeders, (red), male non-breeders (cyan), and female non-breeders (pink).

We provide additional demographic data for another mole-rat species within the *Fukomys* genus (*Fukomys damarensis*). These data were compiled on March 15, 2021, and included lifespans for 702 animals, of which 634 aged past *T*_sex_ and therefore were included in downstream analyses (Supplemental Figure S2A: cyan). These included 502 animals’ lifespans that were censored and 132 deaths. Versus the *F. anselli* data set [34], these data for *F.damarensis* were richer for young and sparser for old ages (Supplemental Figure S2A). Nonetheless, these two species exhibited similar demographic properties, in terms of both Kaplan-Meier (Supplemental Figure S2B) and age-specific mortality hazard (Supplemental Figure S2C). For additional downstream analyses, we combined the data from *F. anselli. *However, with only 2.0% of the population surviving at the time of collection of the final datum (Fig. 5A), it is unlikely that the detected trend was due to observation of an insufficient portion of the genus’ lifespan. And *F. damarensis* into a single data set, representing the *Fukomys* genus (Supplemental Figure S2A: green). The combined *Fukomys* data set included lifespan data from 1041 animals, 966 of which survived past *T*_sex_ (270 days) so were included in downstream analyses; these included 319 deaths and 647 censorships.

Kaplan-Meier survival analysis revealed a *Fukomys* median lifespan of 3622 days (Fig. 5A). Unlike the survival curve for *H. glaber*, the *Fukomys* survival curve approached zero before being depleted of animals, allowing for calculation of a maximal lifespan, as previously defined (95th percentile [14]) of 7369 days (Fig. 5A), or 20.2 years. Age-specific mortality hazard was calculated for *Fukomys*, and again unlike *H. glaber*, substantial increase was observed with age (Fig. 5B). Though less statistically robust, this trend was consistent with estimates taken from either the *F. damarensis* data alone, or from the *F. anselli* data alone, with or without accidental/fighting death censored (Supplemental Figure S2C).

### Mole-rat mortality hazards showed less increase with age than other mammals, with *H. glaber* mortality hazard still uniquely constant

**Fig. 6:**
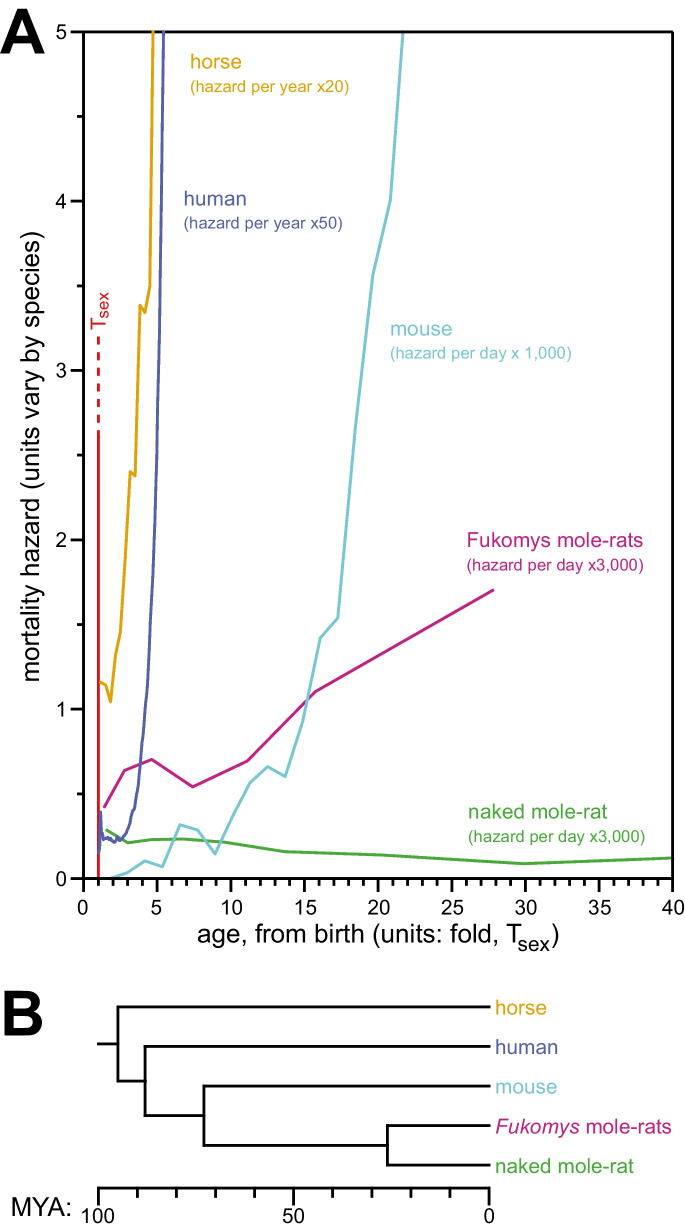
In contrast to the mortality hazards of other mammals, which increased with chronological age, the mortality hazard of naked mole-rats remained constant. **A** Age-specific mortality hazard for humans (navy), mice (cyan), horses (orange), *Fukomys* mole-rats (purple), and naked mole-rats (green); with age (*x*-axis) normalized to *T*_sex_ for each species. Reproduced from (Ruby et al, 2018), with *Fukomys* data added and *H. glaber* data updated using results from this study. See the “Results” for references to original data sources. **B** A phylogenic tree for the species from panel **A**. See the “Results” section for references to data sources.

Age of reproductive maturity (*T*_sex_) for a species is also generally predictive of species MLSP [43–45]. As described previously [14] and shown again here (Fig. 6A), the failure of naked mole-rat mortality hazard to increase with age, even at many-fold the species’ *T*_sex_, is unique among the mammals analyzed. These included laboratory mice (*Mus musculus*) using the raw data for the control mice cohort from the published paper of Miller [46], with *T*_sex_ at 42 days post-natal [47] and a median lifespan of 847 days; humans (*Homo sapiens*), as reported for a 1900 birth cohort [48] and with *T*_sex_ defined as 16 years [49, 50]; and horses (*Equus ferus caballus*), using insurance tables [51] and *T*_sex_ at 3 years [47]. All of these species are anciently diverged from *H. glaber*, with its closest relative being *M. musculus*, that diverged from *H. glaber* ~73 MYA [32]. In contrast, *H. glaber* diverged from humans ~88 MYA and from horses ~95 MYA [52]. These evolutionary relationships are depicted in Fig. 6B.

Here, we added *Fukomys* mole-rat mortality hazard, age normalized to a *T*_sex_ of 270 days, to our comparative mammalian hazard plot (Fig. 6A). *Fukomys* diverged from *H. glaber* 26 MYA (Fig. 6B [16]) and displayed an intermediate rate of hazard increase between the non-increasing hazard of *H. glaber* and the exponentially increasing hazard of other mammals. *Fukomys* mortality hazard appeared to increase linearly, rather than with a Gompertzian exponential. The sparsity of late-life data for that genus made conclusions tentative, as does the uncertainty of age at *T*_sex_. We based our T_sex_ value of 270 days on our own experience, but alternative *T*_sex_ values are reported for *F. anselli* and *F. damarensis* on the AnAge website [47]. However, with only 2.0% of the population surviving at the time of collection of the final datum (Fig. 5A), it is unlikely that the detected trend was due to observation of an insufficient portion of the genus’ lifespan.

## Discussion

### The elusion of demographic aging by *H. glaber* was replicated in an additional 5 years of new data

In this study, we re-visited our analyses and conclusions from our previous paper [14], bringing 5 years’ worth of additional data collection to bear. That period represented a period of unprecedented scale and stability for our collection: while animals continued to be killed for research (KFR) purposes, there were fewer constraints on husbandry resources to require culling of the population. Animals remained at the same institutional vivarium across that period (Calico Life Sciences, LLC), thus avoiding the potentially confounding husbandry challenges of institutional and geographic relocation. Especially given the mature status of the collection across this period (i.e., many animals of advanced age already present), these conditions facilitated an approximate doubling of the available observation numbers for demographic analysis across most age categories (Fig. 2A).

These additional data provided two opportunities: first, to repeat our original analyses on the full, updated historical data, which added resolution and statistical confidence to our original claims (Fig. 1); second, the scale of data collected since our last study facilitated an independent analysis of *H. glaber* lifespan demography, performed entirely on data collected after the completion of data collection for our prior study (navy in Fig. 2). Importantly, that independent analysis validated our original claims about naked mole-rat demography and the absence of a Gompertzian increase in mortality hazard as animals get older.

The form of the age-versus-hazard function can be affected by heterogeneity across the population, in ways that confound inference of a rate-of-aging. In the following, we will describe several scenarios and explain why they were unlikely to confound our analyses.

First: mixed populations with uneven baseline hazards can “flatten” the population-wide plot—as high-hazard individuals die off, they elevate population hazard at young ages; and as those individuals become scarce, the population hazard recedes to that of the lower-baseline individuals at older ages [53]. In our data, the uneven hazard experienced by breeders versus non-breeders (Fig. 3) may indeed contribute to the appearance of reduced hazard with age in the population-wide plot (Fig. 1), albeit due to elimination of the higher-baseline non-breeders more from censorship and conversion into breeders than from mortality. However, independent analysis of these groups continued to exhibit no increase in mortality hazard with age (Fig. 3).

Second: mortality hazard can be uneven across different phases of life, and inclusion of qualitatively distinct life periods can result in inappropriate fits. This concept is well-illustrated by human hazard, which is very high shortly after birth and in early childhood, relatively stable in the early parts of adulthood, then exponentially increasing in mid- and late adulthood [54]. In our analysis of *H. glaber*, 'childhood' mortality (prior to *T*_sex_) was excluded, and hazard was only slightly elevated in the earliest adulthood bin (183–400 days); beyond that, hazard was similarly low across subsequent, independently analyzed bins (Fig. 1). Our independent analysis of each age bin limited the opportunity for life-phase changes to impact our conclusions via a functional fit over an inappropriately dilated age range.

Third: extending the second scenario, dealing with different demographic phases of life, it has been argued that our demographic data spanning over three decades of age is insufficient to characterize mortality hazard as non-increasing (public reviews [55]). Below, we explain how fundamental principles from the fields of demographic statistics and biological aging address those objections. We highlight the potential causes of disparate mortality hazard at different life stages, both in the wild versus captivity and under suboptimal husbandry conditions.

Regarding different life stages: the Makeham component of human demographic aging corresponds to age-independent mortality, often described as “accidental death,” which obscures age-dependent mortality at young ages [56]. The Makeham stage is not biologically or demographically distinct, and its extent is proportional to the level of accidental hazard; it can only hide age-dependent hazard so long as the Makeham stage is substantially greater. The extended period across which *H. glaber* hazard was observed to be approximately constant (Fig. 1) as well as its very low value when compared to that of other captive species (Fig. 6) severely constrains its ability to hide a hypothetical age-dependent hazard component for this species.

Regarding the sufficiency of our longitudinal data: it has been argued that over three decades of observed non-increasing mortality hazard is insufficient for any demographic analysis due to only ~50% of the survival curve having been achieved (public reviews [55]). We counter that premise, on the basis of the readily apparent age-dependent increase in mortality hazard prior to median survival for the other species examined: lab mice, humans, and horses (Fig. 6). We further point out that the large surviving fraction of the population after 30+ years resulted from our ability to maintain a very low baseline mortality rate in our captive population. Had our animal care been worse, then our observation of constant-with-age hazard would have extended further down the survival curve, and again, our low baseline hazard reduced the Makeham component’s ability to mask age-related hazard. Finally, we point out that, according to the allometric scaling rules [10], *H. glaber* should have a maximum lifespan of 8 years; and in the wild, non-breeding members of this species only remain in the their natal colony for a maximum of 4 years [5]. Thus, the lack of any evidence of demographic aging, even at fivefold or more the expected or real-world maximum survival, seems incompatible with a yet-to-be-observed biological life phase in which hazard increases.

### Elevated hazard for non-breeders in small colonies

We previously reported that breeders of both sexes of naked mole-rats have substantially lower mortality hazards than non-breeders at all ages [14], and that result was reproduced here (Fig. 3). This result was not limited to *H. glaber*: our analysis of *F. damarensis* survival also revealed an advantage for breeders over non-breeders. This trait is shared with many cooperative breeding mammals [57–59] and is also similar to findings of increased longevity in the breeding queens of the social hymenopterans, where the queens outlive non-reproductive workers by 50–100-fold [60, 61], possibly suggesting that it is reproduction itself that delays aging. These observations conflict with the disposable soma theory of aging [62] but do not negate it: there are also multiple biological and sociological explanations for the survival advantage of socially dominant individuals (e.g., [63–67]) that may confound one another and variably contribute to the fates of breeders versus non-breeders. It is possible that the higher mortality of non-breeders in captivity is linked to chronic stress, being unable to escape the bullying from the dominant breeding animals [68, 69]. It is, however, unlikely that the disparate mortality risk of breeders versus non-breeders is due to the fact that breeders are of “higher quality.” In captivity in colonies where the queen dies and animals fight to death to establish dominance, the queens within the colony may be of “higher quality,” which may contribute to their mortality hazard. However, most of our breeders are randomly chosen, in that we take out animals from colonies to pair and cannot necessarily choose animals of better somatic quality.

In order to gain deeper insight into the health consequences of naked mole-rat social hierarchy amongst non-breeders, we analyzed historical records of body weight and colony membership in terms of their impacts on mortality hazard (Fig. 4). Dominance in naked mole-rat colonies is largely determined by aggressive pushing, tail-tugging, and shoving behaviors, so smaller animals are generally lower in the social hierarchy than larger [63, 69, 70]. It was therefore unsurprising to observe that smaller adult animals had higher mortality hazard than bigger animals (Fig. 4C), though that trend did not attain statistical significance in our analysis.

More surprising was the observation that non-breeders who were members of large colonies had lower mortality hazard than non-breeders who were members of small colonies, an observation that was statistically significant for both sexes (Fig. 4E). One might have expected fewer colony members to result in less aggressive competition for, and therefore better access to, limited resources, e.g., food and heating pads [71]. However, such concerns may have been less relevant to our captive colonies than in the wild: animal husbandry rules necessitate the scaling up of all resources in parallel with colony growth [40]. Further, even in the wild, intra-group competition is often trumped by cooperative advantages to produce overall survival advantages for members of larger social groups across a variety of species [72–75]. Larger colonies can also provide greater stability, with a well-established successfully breeding female and a cohort of older siblings having already resolved their dominance status and keeping the younger animals in check [40].

### The mortality hazard of Fukomys mole-rats increased gradually with age

We sought to provide new evolutionary context for the exceptional longevity of naked mole-rats through demographic analysis of mole-rats from the *Fukomys* genus. Though having substantially diverged from *H. glaber* 26 MYA [16], *Fukomys* mole-rats offered a phylogenetically closer comparator versus other mammals for which data are available (Fig. 6B). Further, *Fukomys* mole-rats share the property of eusociality with naked mole-rats, which is thought to be connected to exceptional lifespan by evolutionary pressures [16, 32]. Demographic data were published for Ansell’s mole-rat, *F. anselli* [34]; we supplemented those data with lifespan data from the closely related Damaraland mole-rat, *F. damarensis*. Those two species are estimated to have diverged ~1–1.5 MYA [76], and they shared a similar ecological niche in the wild and statistically consistent life history patterns in captivity (Supp Figure S2). We combined the data from these species for a *Fukomys* genus-wide demography analysis and observed hazard to increase with age (Fig. 5B).

While the increase of mortality hazard with age among *Fukomys* mole-rats contrasted with the age-independent hazard of *H. glaber*, it was gradual versus the Gompertzian (i.e., exponential) increase typically exhibited by mammals (Fig. 6A). This linear shape of the *Fukomys* mortality risk with advancing years may possibly reflect an increase in early-life mortality due to “less-than-ideal” husbandry conditions for these species such that as these conditions are optimized for the species a more robust exponential Gompertzian relationship may manifest. Although we cannot speak for the husbandry of *F.anselli*, using breeding success as a metric of optimal husbandry, our Damaraland colonies breed well in captivity. Moreover, many individuals in our care have now attained ages way beyond that predicted on the basis of body size. At ages approximating double their expected maximum lifespan, their fur starts graying, especially around the face; however, to date, there are no published reports of age-associated physiological changes or pathology [77]. As such, we do not feel that suboptimal husbandry is the cause of this unusual relationship between mortality risk and age.

Regarding the possibility, raised earlier in the “Discussion” section, of missing demographic aging in *H. glaber* due to less-than-ideal husbandry conditions: it is worth noting that we have achieved lower mortality rates for *H. glaber* than for *Fukomys* in captivity, and even with higher extrinsic mortality, hazard increase with age is still readily observable in *Fukomys*, with substantially shorter observation time than for *H. glaber*, in terms of both actual and *T*_sex_-adjusted age (Figs. 5 and 6, S2).

Various evolutionary theories highlight multiple aspects of mole-rat biology and lifestyle that could either drive or derive from the evolution of such extended longevity. Subterranean rodents, regardless of clade or whether they are solitary or social, live longer than expected for their size, with environmental protection from extrinsic mortality [5, 78]. Similar protection from extrinsic mortality is also provided by herd protection/social lifestyle [5, 78]. Low extrinsic hazards give evolutionary tinkering with aging rates the opportunity to manifest selective advantage [79], a premise backed up by several cross-species studies [80]. In the context of eusociality, kin selection and the maintenance of stable social hierarchies can provide further selective advantages to the extension of lifespan for breeders [63–66]. The converse may also be true, with enhanced longevity facilitating a eusocial lifestyle [81]: increased longevity may facilitate the cohabiting of parents and multiple litters of offspring to form a eusocial colony, enhancing mutual beneficial opportunities for cooperative living and overall fitness of the social group [60, 61].

Regardless of which evolutionary theory best applies to the natural history of *H. glaber* and *Fukomys* mole-rats, their shared (though unequal) defiance of Gompertzian aging demonstrates that exponentially increasing hazard with advancing age is not a fixed property of mammalian biology. While it seems to be the default, evolutionary mechanisms have managed to dampen or eliminate it when provided with the right selective incentives. It is not clear the extent to which both *H. glaber* and *Fukomys* benefit from longevity genes that arose in their common ancestor, versus evolved independent longevity-promoting mechanisms in the 26 million years since their divergence. However, their unequal successes suggest that multiple mechanisms are needed to counteract Gompertzian aging, and that not all mechanisms are at play in both lineages. The perspective that some age-slowing mechanisms are likely to be conserved between *H. glaber* and *Fukomys*, and that others are likely to be specific to *H. glaber*, should help the comparative biology community make maximal use of these two valuable natural resources for the science of aging.

### Supplementary information

ESM 1 (PDF 510 kb)

ESM 2 (PDF 542 kb)

ESM 3 (XLSX 29 kb)

ESM 4 (XLSX 311 kb)

ESM 5 (XLSX 328 kb)

ESM 6 (XLSX 39 kb)
