# Supplementary figures and images for: Five years later, with double the demographic data, naked mole-rat mortality rates continue to defy Gompertzian laws by not increasing with age
Source: GeroScience. 2024 May 21;46(5):5321–41. doi: 10.1007/s11357-024-01201-4 (PMC11336006; doi:10.1007/s11357-024-01201-4)

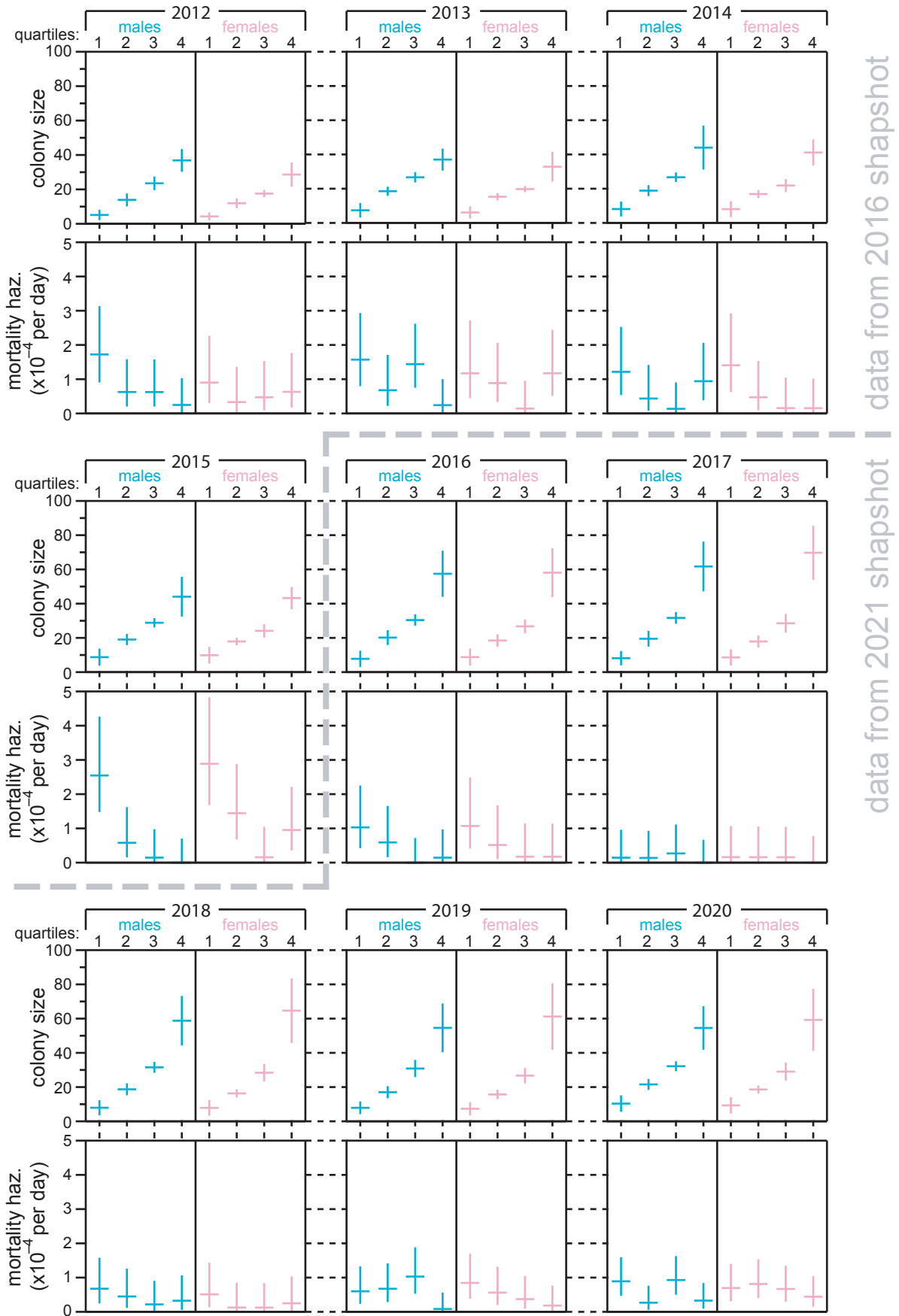

Supplement: Supplementary file 1 — (PDF 510 kb) [file 11357_2024_1201_MOESM1_ESM.pdf]

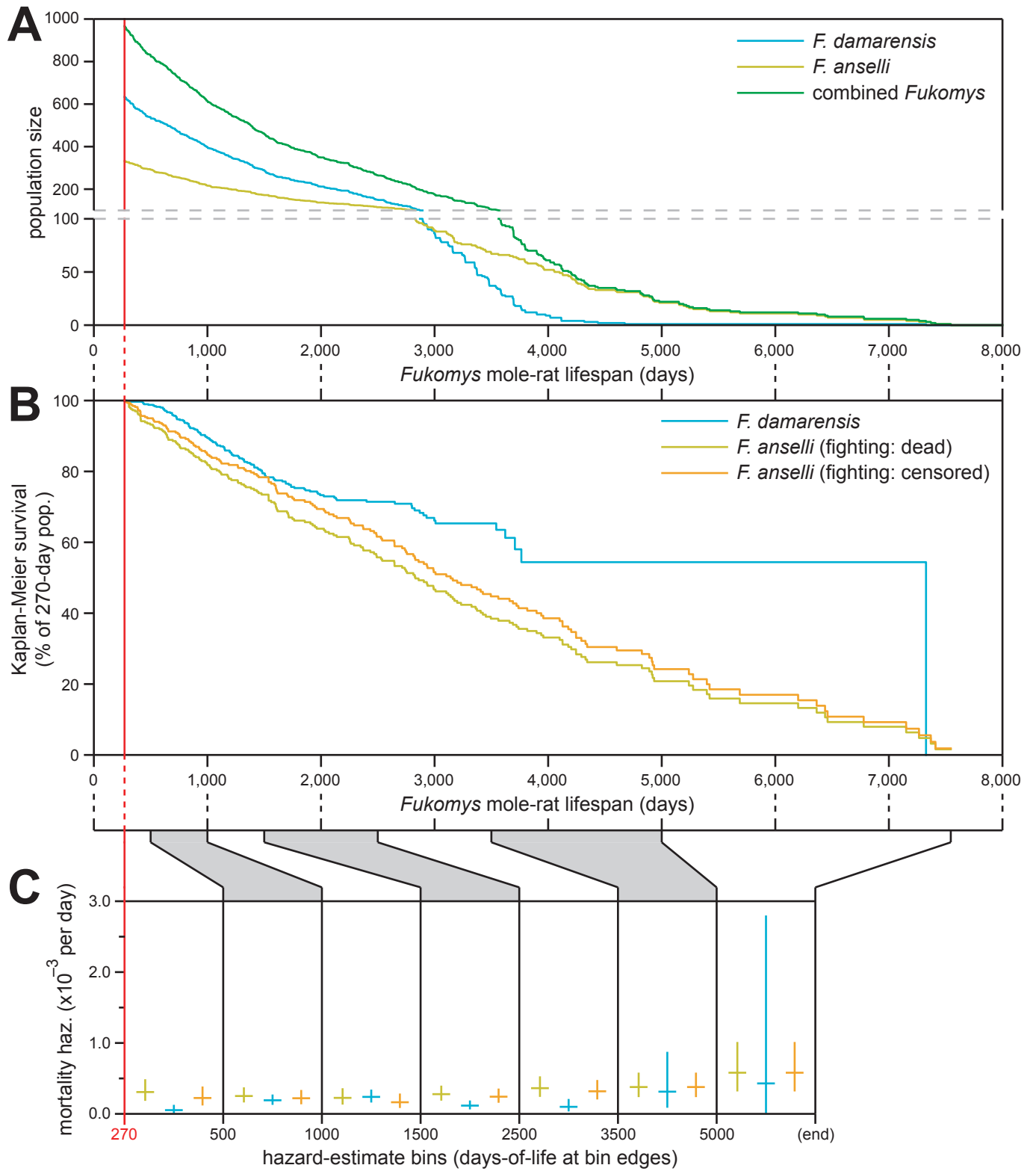

Supplement: Supplementary file 2 — (PDF 542 kb) [file 11357_2024_1201_MOESM2_ESM.pdf]
